# Impact of Gross Strap Muscle Invasion on Outcome of Differentiated Thyroid Cancer: Systematic Review and Meta-Analysis

**DOI:** 10.3389/fonc.2020.01687

**Published:** 2020-09-25

**Authors:** Li Zhang, Jia Liu, Peisong Wang, Shuai Xue, Jie Li, Guang Chen

**Affiliations:** ^1^Department of Nephrology, The First Hospital of Jilin University, Changchun, China; ^2^Department of Thyroid Surgery, The First Hospital of Jilin University, Changchun, China; ^3^Department of Geriatric, The First Hospital of Jilin University, Changchun, China

**Keywords:** strap muscle invasion, prognosis, differentiated thyroid carcinoma, meta-analysis, review

## Abstract

**Background:** Gross strap muscle invasion (gSMI) in patients with differentiated thyroid cancer (DTC) was defined as high-risk recurrent group in the 2015 American Thyroid Association guidelines. However, controversy persists because several studies suggested gSMI had little effect on disease outcome. Herein, a systematic review and meta-analysis was conducted to investigate impact of gSMI on outcome of DTC.

**Methods:** A systematic search of electronic databases (PubMed, EMBASE, Cochrane Library, and MEDLINE) for studies published until February 2020 was performed. Case-control studies and randomized controlled trials that studied the impact of gSMI on outcome of DTC were included.

**Results:** Six studies (all retrospective studies) involving 13,639 patients met final inclusion criteria. Compared with no extrathyroidal extension (ETE), patients with gSMI were associated with increased risk of recurrence (*P* = 0.0004, OR, 1.46; 95% CI: 1.18–1.80) and lymph node metastasis (LNM) (*P* < 0.00001, OR 4.19; 95% CI: 2.53–6.96). For mortality (*P* = 0.34, OR 1.47; 95% CI: 0.67–3.25), 10 year disease-specific survival (*P* = 0.80, OR 0.91; 95% CI: 0.44–1.88), and distant metastasis (DM) (*P* = 0.21, OR 2.94; 95% CI: 0.54–15.93), there was no significant difference between gSMI and no ETE group. In contrast with maximal ETE(extension of the primary tumor to the trachea, esophagus, recurrent laryngeal nerve, larynx, subcutaneous soft tissue, skin, internal jugular vein, or carotid artery), patients with gSMI were associated with decreased risk of recurrence (*P* < 0.0001, OR, 0.58; 95% CI: 0.44–0.76), mortality (*P* = 0.0003, OR 0.20; 95% CI: 0.08–0.48), LNM (*P* = 0.0003, OR 0.64; 95% CI: 0.50–0.81), and DM (*P* = 0.0009, OR 0.28; 95% CI: 0.13–0.59).

**Conclusions :** DTC patients with gSMI had a higher risk of recurrence and LNM than those without ETE. However, in contrast with maximal ETE, a much better prognosis was observed in DTC patients with only gSMI.

## Introduction

Extrathyroidal extension (ETE), which is defined as tumor spread outside of the thyroid gland and into the surrounding tissues, occurs in up to 30% of patients with differentiated thyroid cancer (DTC) (1, 2). Minimal ETE (mETE), detectable only on histological examination, was not regarded as a negative predictor for either survival or disease recurrence (3–5). Accordingly, mETE was removed from the T3 definition in the 8th edition of the American Joint Committee on Cancer (AJCC) classification, as it would not affect either T category or overall stage (6). In contrast, gross ETE is believed to be an important risk factor for recurrence and mortality (7, 8). Thus, DTC patients with gross ETE are classified as T3b or T4 in the AJCC system (9). Moreover, the 2015 American Thyroid Association (ATA) guidelines grouped tumors with gross ETE in the high risk of recurrence category, with a nearly 20% risk of structural recurrence (10). Therefore, gross ETE was an absolute indication for total thyroidectomy and the administration of post-operative radioactive iodine.

In addition to the degree of gross ETE, the site of gross tumor invasion also plays important roles in disease-specific survival (DSS) and disease-free survival (DFS). Recently, several studies reported that gross strap muscle invasion (gSMI) had little effect on DSS and DFS, which was different from the findings of previous studies (8, 11). Increasing evidence suggests that DTC patients with only gSMI have the same DFS as those with microscopic ETE (11). In our previous study, we also found that only four of 30 (13.3%) Braf-mutated thyroid papillary microcarcinoma patients with gSMI were diagnosed with recurrence (12). Accordingly, Shaha (13) suggested that a detailed distinction of gross ETE should be performed. Patients with anterior ETE involving the strap muscle had a relatively good prognosis compared with those with posterior gross ETE to the recurrent laryngeal nerve, trachea or esophagus (8). A possible reason is that gSMI can be easily resected with negative margins (13).

In light of the conflicting data on the recurrence risk and mortality conferred by gSMI, we performed a systematic review and meta-analysis to assess the impact of gSMI on the outcomes of DTC patients.

## Materials and Methods

This meta-analysis was conducted in accordance with the Cochrane Handbook for Systematic Reviews of Interventions guidelines (14). There was no funding received for this study.

### Search Strategy

We searched the EMBASE, PubMed, MEDLINE, and Cochrane databases from inception to February 26, 2020, without any language limitations. The following terms were used in the search: “thyrohyoid muscle,” “sternothyroid muscle,” “sternohyoid muscle,” “strap muscle,” “gross extrathyroidal extension,” “invasion,” “extension,” “thyroid cancer^*^,” “thyroid carcinoma^*^,” and “thyroid neoplasm^*^.”

### Inclusion and Exclusion Criteria

The studies returned from the search were checked according to the following inclusion criteria: ① patients were more than 18 years old; ② pathologically proven DTC patients who underwent surgery; ③ complete clinical data and follow-up information; and ④ DTC patients with SMI.

The exclusion criteria were as follows: ① patients with <12 months of follow-up; ② those with incomplete medical records; ③ those with medullary thyroid carcinoma and undifferentiated carcinoma; and ④ publication styles were letters to the editor, abstracts and meeting posters.

### Data Extraction and Risk of Bias Assessment

L. Zhang and J. Liu assessed the search results according to the relevance in providing information for the review. Two reviewers (L. Zhang and S. Xue) independently assessed the titles and abstracts of the remaining records for relevance according to the protocol criteria. Then, they browsed the full text of the studies in detail. Any disagreements were resolved by consulting a third reviewer (J. Li). L. Zhang assessed the risk of bias of each included study using the relevant, validated tool for each study design. J. Liu performed the risk of bias assessment. The risk of bias of the included trials was assessed using the Newcastle-Ottawa Scale (15).

### Statistical Analysis

Review Manager (RevMan) 5.3 software was used for the analysis. We calculated odds ratios (ORs) with 95% confidence intervals (CIs) for dichotomous data. We assessed the heterogeneity across studies using the *Q*-test and the *I*^2^ statistic. *P* < 0.1 and *I*^2^ > 50% indicated statistical significance (16). If there was obvious heterogeneity, we used a random-effects model; otherwise, we used a fixed-effects model. We conducted sensitivity analysis by excluding each single study at a time to test its influence on the pooled effects. The source of heterogeneity was also explored by subgroup analyses of operation type and histopathological subtype based on available information. When the *p*-value was < 0.05, it was considered statistically significant.

## Results

### Literature Search

**Figure 1:**
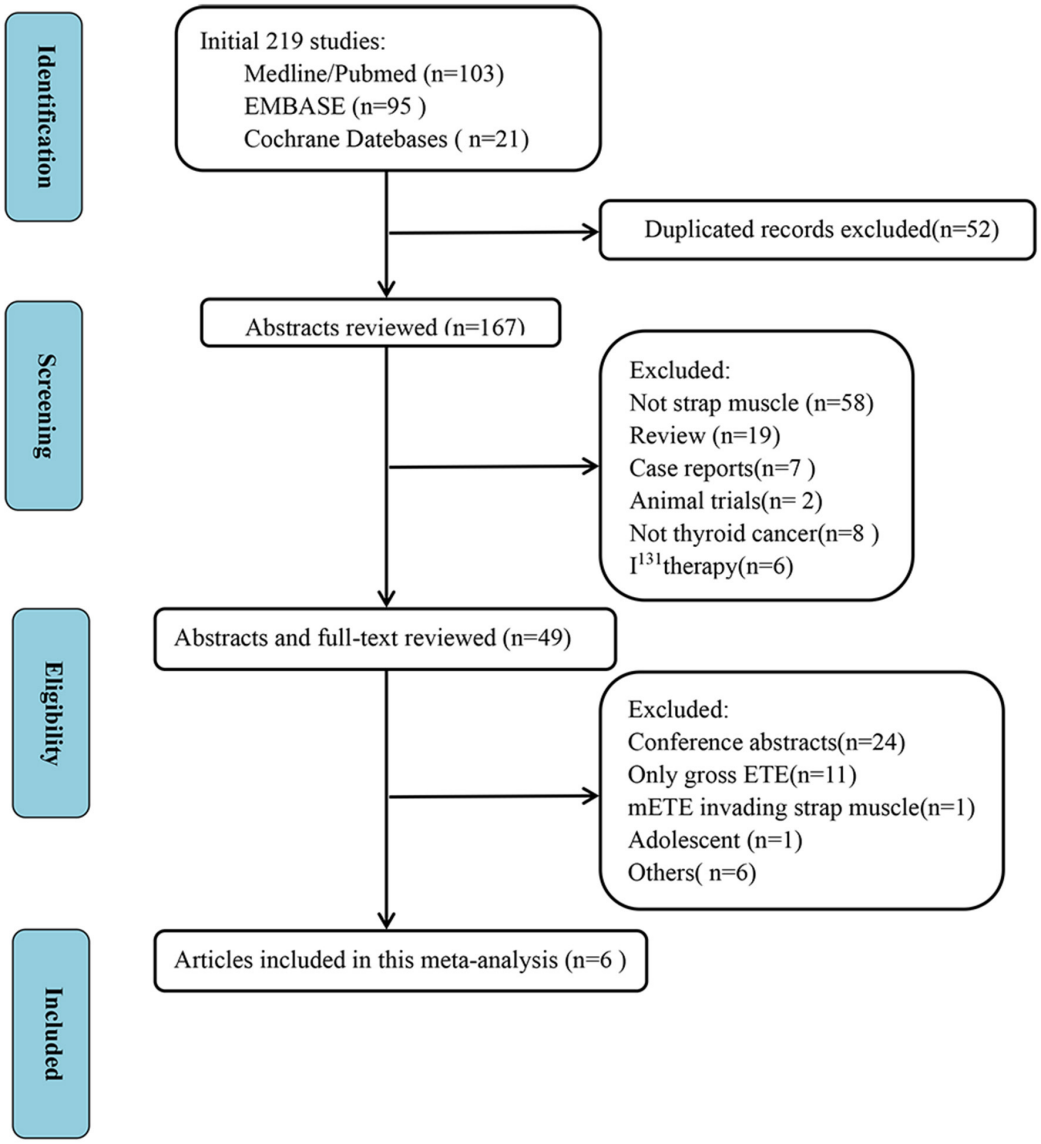
Selection process of studies in meta-analysis.

We initially identified a total of 219 studies. Fifty-two duplicate studies and another 118 studies were excluded after reviewing the titles and abstracts. After scrutiny of the full texts of the remaining 49 articles, six studies were finally included in this meta-analysis, all of which were retrospective studies (8, 11, 17–20). Figure 1 shows the study selection process.

### Study Characteristics and Quality

In this meta-analysis, a total of 13,639 patients were included, and the characteristics of the included studies are presented in Table 1. The maximal ETE means extension of the primary tumor to the trachea, esophagus, recurrent laryngeal nerve, larynx, subcutaneous soft tissue, skin, internal jugular vein, or carotid artery. No ETE means no extrathyroidal extension. The quality assessment of the included studies by the Newcastle-Ottawa Scale is presented in Table 2. All studies used hospital controls, who were accessed by the same method as gross ETE into the strap muscle (gETE st+ group). Multivariate analysis was conducted by all the studies. The scores of all the studies were over 5; thus, the quality of the selected studies was generally high.

**Table 1:** Baseline characteristics of included studies.

**Study**	**Patients no**.	**Groups**	**Patient no**.	**Female (n, %)**	**Age (y) Mean (SD)/Median (range)**	**Tumor size (mm) Mean (SD)/Median (range)**	**RAI (*n*, %)**	**Multifocality (*n*, %)**	**N stage**	**Follow-up Mean (SD)/Median (range)**
Amit et al. (11)	2,084	No ETE (group 1)	1,291	966 (75)	47 (18–87)	N/A	555 (44)	479 (37)	Nx	66 (12–192)
		ETE into perithyroidal soft tissue (group 2)	732	527 (72)	47 (18–97)		527 (74)	397 (54)		51 (12–189)
		gETE into strap muscle (group 3)	61	52 (85)	48 (19–83)		48 (78)	29 (48)		64 (17–155)
Li et al. (18)	4,045	No ETE	2,300	1,679 (73.0)	42.6 (10.5)	11.17 (8.11)	928 (40.3)	625 (27.1)	N/A	30 (12–63)
		ETE into perithyroidal tissue	1,004	726 (72.3)	43.2 (12.1)	13.64 (9.61)	634 (63.2)	274 (27.3)		34 (12–63)
		T3b (gETE into the strap muscles)	371	286 (77.1)	44.1 (10.4)	18.42 (10.61)	325 (87.6)	124 (33.4)		32 (13–58)
		ETE beyond the strap muscles	370	262 (70.8)	46.3 (11.1)	21.15 (9.88)	361 (97.6)	134 (36.2)		30 (12–59)
Park et al. (19)	3,174	No ETE	1,362	1,170 (85.9)	45.5 (11.6)	12.5 (11.0)	1,024 (75.2)	N/A	N0 N1	148.8 (133.2–174)
		Microscopic ETE	1,377	1,199 (87.1)	46.2 (12.2)	15.3 (11.9)	1,299 (94.3)			
		gETE invading only strap muscles	261	227 (87.0)	49.0 (13.1)	19.1 (13.5)	246 (94.3)			
		gETE invading perithyroidal structures	174	150 (86.2)	52.4 (15.0)	24.2 (18.7)	153 (87.9)			
Song et al. (8)	3,104	T1	1,997	2,712 (87.4)	45.9 (37.8–54.2)	13 (8–22)	2,363 (76.1)	N/A	N0 N1	120 (97.2–144)
		T2	496							
		Y3a	96							
		T3b(≤ 4 cm and gETE to strap muscle)	376							
		T3b(>4 cm and gETE to strap muscle)	38							
		T4a	101							
Song et al. (20)	636	Without gETE	586	457 (78.0)	45.0 (11.4)	12 (10–15)	N/A	53 (9.0)	N0 N1a	91.2 (61.2–130.8)
		Those with gETE to strap muscle	50	45 (90.0)	49.9 (8.9)	12 (11–15)		7 (14.0)		84 (60–102)
Danilovic et al. (17)	596	Low-risk PTC without ETE (low w/o ETE)	251	231 (92)	50.8 (12)	14.9 (16.4)	102 (40.6)	107 (43)	N0 N1a	48 (12–322)
		Intermediate-risk PTC without ETE (intermediate w/o ETE)	89	76 (85.4)	44.9 (15.8)	24.1 (18.9)	81 (91)	57 (64.8)		43.2 (12–217)
		Minimal(mETE)	191	166 (86.9)	50.0 (13.7)	17.3 (13.4)	186 (97.4)	121 (63.4)		39.6 (12–142)
		gETE into the strap muscles(gETE)	65	54 (83.1)	51.6 (14.5)	28.0 (16.0)	64 (98.5)	35 (54.7)		43.2 (12–260)

*N/A, not applicable; SD, standard deviation; ETE, extrathyroidal extension; gETE, gross extrathyroidal extension; RAI, radioactive iodine; LN, lymph node; RLN, recurrent laryngeal nerve*.

**Table 2:** Quality assessment of the included studies by the Newcastle-Ottawa Scale (score).

**Items**	**Amit (11)**	**Li (18)**	**Park et al. (19)**	**Song (8)**	**Song (20)**	**Danilovic (17)**
**Selection**						
Is the case definition adequate?	1	1	1	1	1	1
Representativeness of the cases	1	1	1	1	1	1
Selection of controls	0	0	0	0	0	0
Definition of controls	1	1	1	1	1	1
Comparability of cases and controls on the basis of the design or analysis	2	2	2	2	2	2
**Outcome**						
Ascertainment of exposure	1	1	1	1	1	1
Same method of ascertainment for cases and controls	1	1	1	1	1	1
Non-response rate	0	0	1	0	0	0
**Total score**	7	7	8	7	7	7

### Outcomes

#### Locoregional Recurrence (LRR)

**Figure 2:**
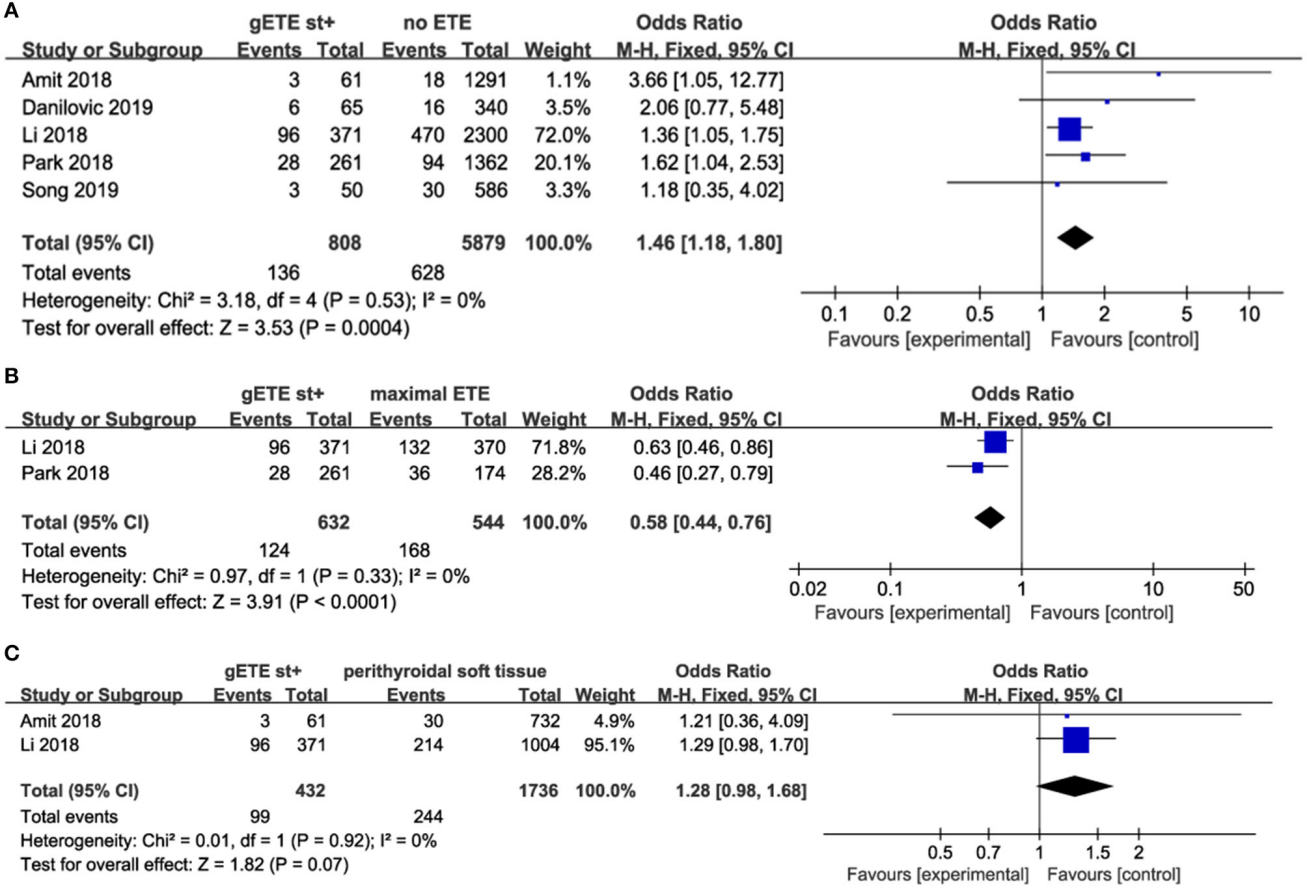
Forest plot for LRR comparison between gETE st+ with **(A)** no ETE **(B)** maximal ETE **(C)** perithyroidal soft tissue.

Five studies evaluated the impact of gSMI on recurrence in 6,687 patients with DTC (no ETE in 5,879 subjects, gETE st+ in 808 subjects). gSMI in patients was associated with an increased risk of recurrence (*P* = 0.0004; OR, 1.46; 95% CI: 1.18–1.80) without heterogeneity (*I*^2^ = 0%) (Figure 2A). Two studies compared the impact of gSMI on recurrence with maximal ETE. Compared with maximal ETE, gSMI was associated with a decreased risk of recurrence (*P* < 0.0001; OR, 0.58; 95% CI: 0.44–0.76) without heterogeneity (*I*^2^ = 0%) (Figure 2B). At the same time, two studies compared the impact of gSMI on recurrence with perithyroidal soft tissue invasion. The locoregional recurrence between patients with gSMI and those with perithyroidal soft tissue invasion was not significantly different (*P* = 0.07; OR, 1.28; 95% CI: 0.98–1.68) without heterogeneity (*I*^2^ = 0%) (Figure 2C).

#### Overall Mortality

**Figure 3:**
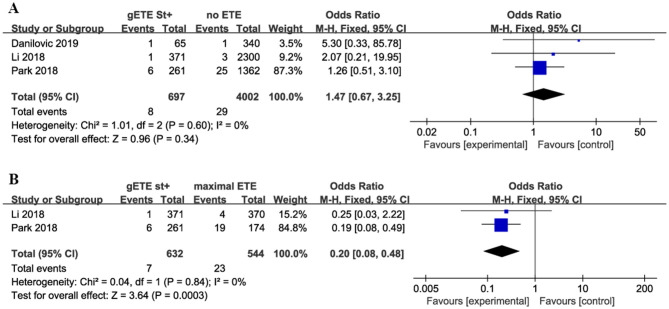
Forest plot for overall mortality comparison between gETE st+ with **(A)** no ETE **(B)** maximal ETE.

Three studies compared the impact of gSMI on cancer-related mortality with no ETE group among 4,699 patients with DTC (no ETE in 4,002 subjects, gETE st+ in 697 subjects). Two studies compared the impact of gSMI on cancer-related mortality with the maximal ETE group. The mortality of patients with gSMI was not increased compared with that of no ETE patients (*P* = 0.34; OR, 1.47; 95% CI: 0.67–3.25) (Figure 3A). Compared with maximal ETE, gSMI was associated with decreased mortality (*P* = 0.0003; OR, 0.20; 95% CI: 0.08–0.48) (Figure 3B).

#### 10 Year Disease-Specific Survival

**Figure 4:**
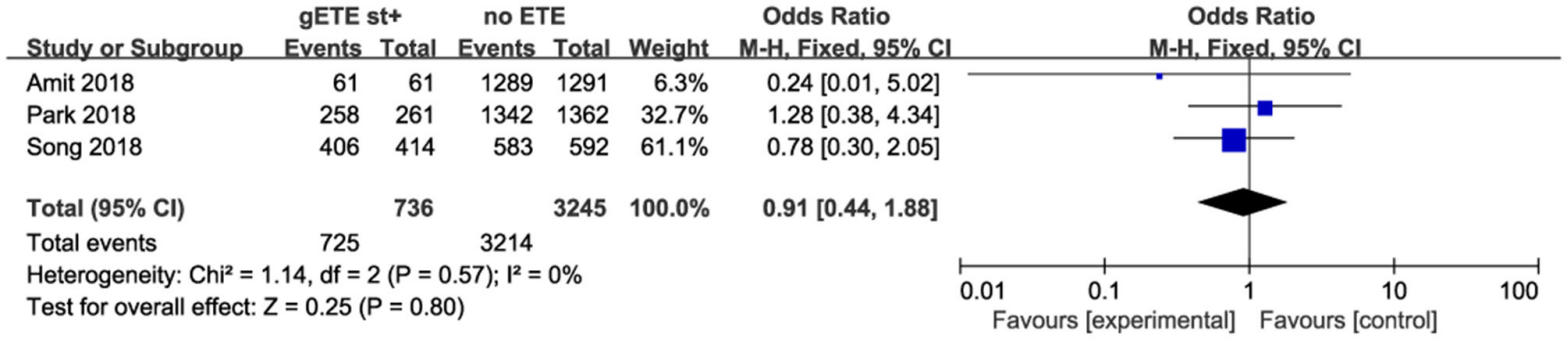
Forest plot for 10 year DSS comparison between gETE st+ with no ETE.

Three studies analyzed the impact of gSMI and no ETE on 10 year DSS among 3,981 patients with DTC. There was no significant difference between the no ETE and gSMI groups (*P* = 0.80; OR, 0.91; 95% CI: 0.44–1.88) with no heterogeneity (*I*^2^ = 0%) (Figure 4).

#### Lymph Node Metastases

**Figure 5:**
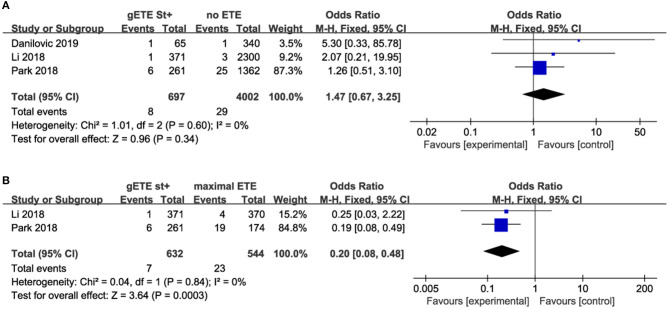
Forest plot for LNM between gETE st+ with **(A)** no ETE **(B)** maximal ETE.

Four studies compared the impact of gSMI and no ETE on baseline lymph node metastases (LNM) among 6,051 patients. gSMI was associated with an elevated LNM ratio (*P* < 0.00001; OR, 4.19; 95% CI: 2.53–6.96) with significant heterogeneity (*I*^2^ = 86%) (Figure 5A). Moreover, only two studies investigated the impact of gSMI and maximal ETE on LNM among 1,176 patients. Compared with maximal ETE, gSMI in patients was associated with decreased LNM (*P* = 0.0003; OR, 0.64; 95% CI: 0.50–0.81) (Figure 5B).

#### Distant Metastases

**Figure 6:**
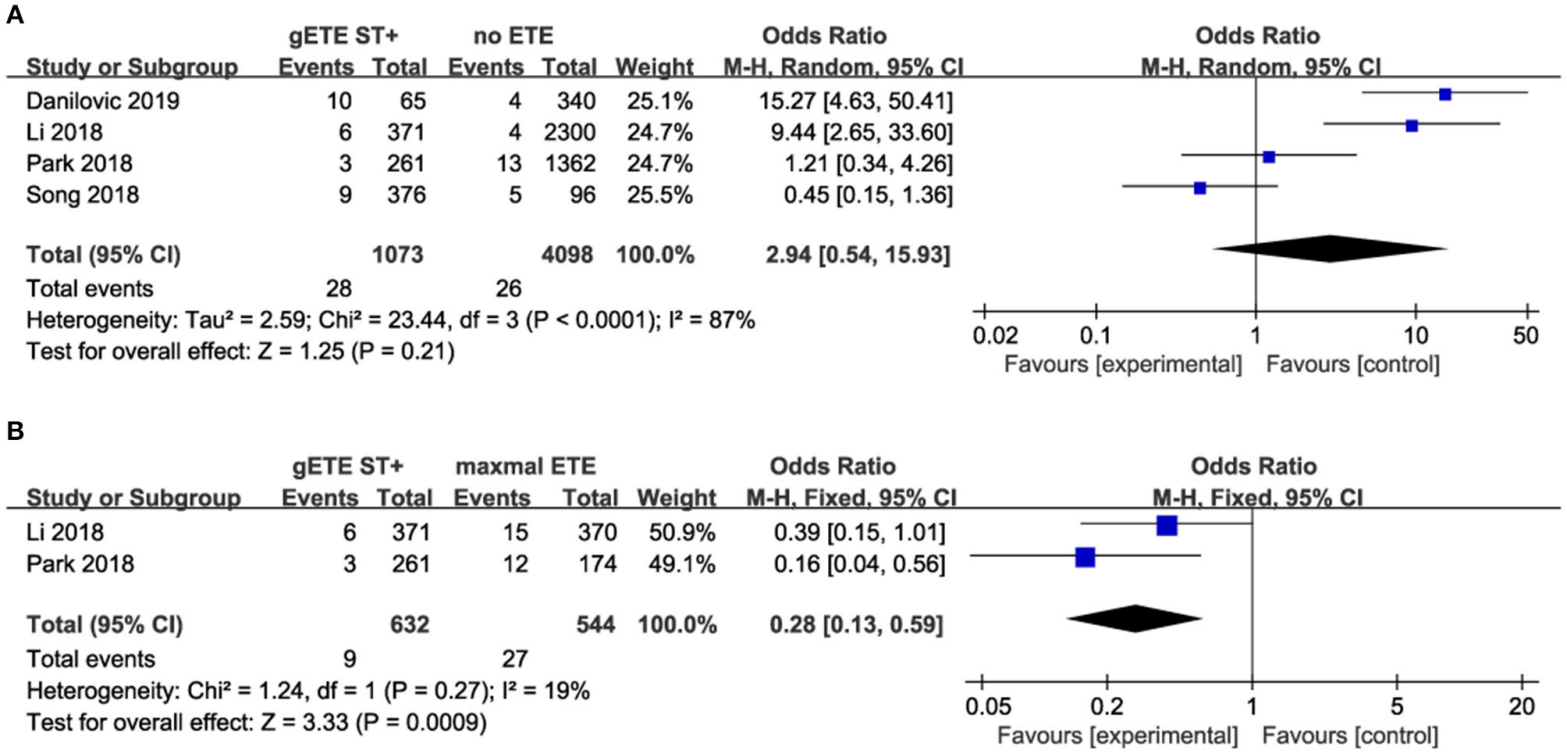
Forest plot for DM comparison between gETE st+ with **(A)** no ETE **(B)** maximal ETE.

Four studies assessed the impact of gSMI and no ETE on distant metastases (DM). There was no significant difference in the DM ratio between the gSMI and no ETE groups (*P* = 0.21), with significant heterogeneity (*I*^2^ = 87%) (Figure 6A). Moreover, only two studies investigated the impact of gSMI and maximal ETE on DM. gSMI in patients was associated with decreased DM (*P* = 0.0009; OR, 0.28; 95% CI: 0.13–0.59) with no heterogeneity (*I*^2^ = 19%) (Figure 6B).

### Sensitivity Analysis and Subgroup Analysis

For the comparisons with significant heterogeneity, we conducted sensitivity analysis. The leave-one-out meta-analysis revealed that LNM (compared with no ETE) and DM (compared with no ETE) did not identify a single study that may have caused the substantial heterogeneity (Data not shown).

**Figure 7:**
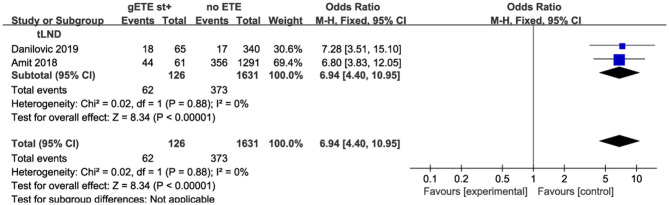
Subgroup analysis for LNM comparison between gETE st+ with no ETE.

Furthermore, the degree of LNM is highly dependent on the type of lymph node dissection (LND). The heterogeneity could derive from the fact that data of the literature generally do not allow differentiation between nodal involvement in the central and in the lateral compartment. Indeed, CLND may be selectively performed whereas lateral neck is usually treated only with a therapeutic intent. Prophylactic LND will identify many microscopic LNM, while therapeutic LND is only performed for patients with clinical metastatic lymph nodes. All and some patients underwent prophylactic LND in the Li and Park studies, respectively. Therapeutic LND was performed in the studies by Danilovic and Amit. In the therapeutic LND subgroup, patients with gSMI had increased LNM compared with patients without ETE (*P* < 0.00001; OR, 6.94; 95% CI: 4.40–10.95; *I*^2^ = 0%) (Figure 7).

**Figure 8:**
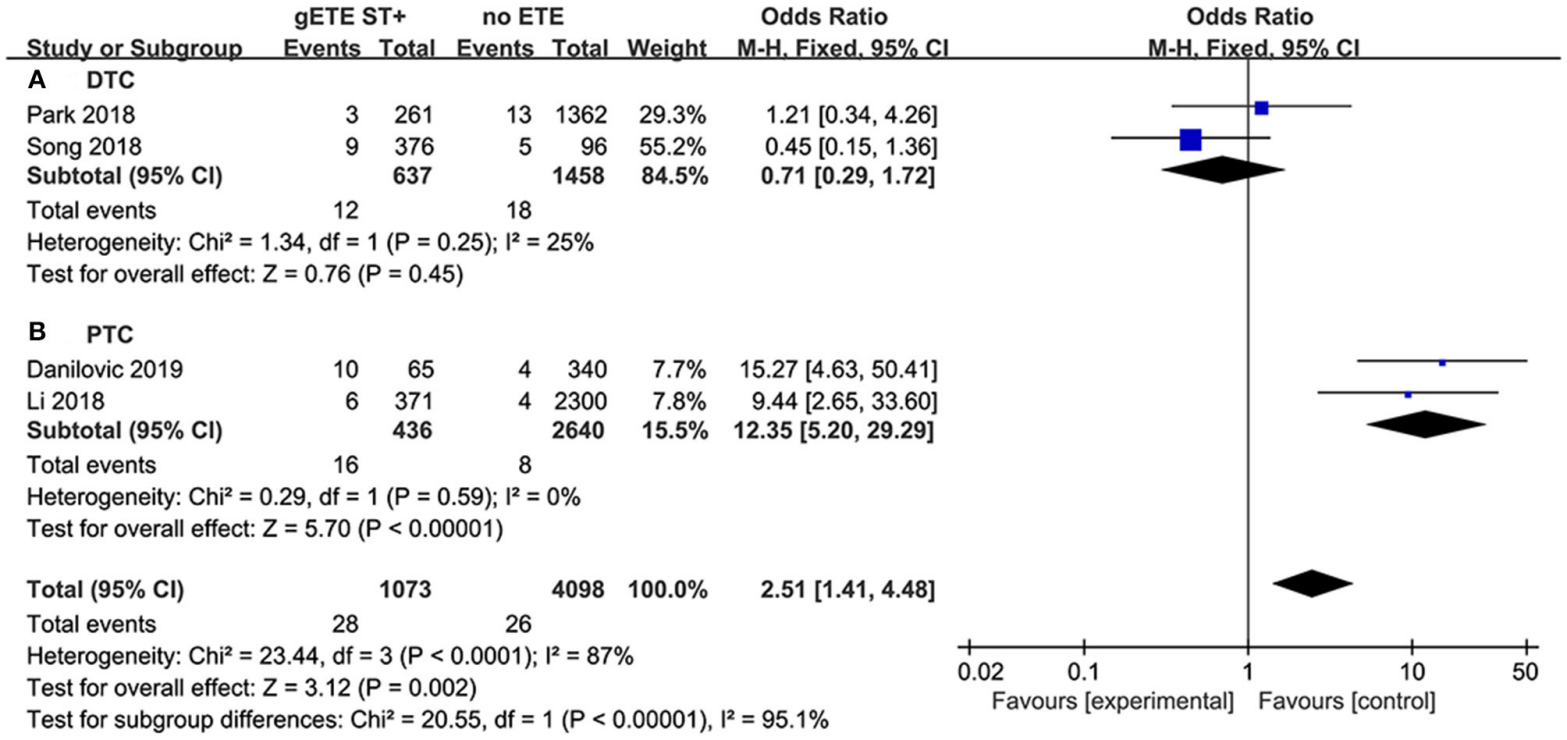
Subgroup analysis for DM comparison between gETE st+ with no ETE in DTC **(A)** and PTC **(B)**.

Patients with follicular thyroid carcinoma are more likely to present with DM than those with papillary thyroid carcinoma (PTC). We believe the significant heterogeneity of the DM analysis is mainly attributed to histopathological types. In the DTC subgroup, there was still no significant difference in DM between the gSMI and no ETE groups (*P* = 0.45) without heterogeneity (*I*^2^ = 25%) (Figure 8A). However, in the PTC subgroup, we found that gSMI increased DM significantly compared with no ETE (*P* < 0.0001; OR, 12.35; 95% CI: 5.20–29.29; *I*^2^ = 0%) (Figure 8B).

## Discussion

Increasing evidence has shown that the site of gross tumor invasion also plays important roles in the recurrence and mortality of DTC patients (21, 22). Some researchers believed that gSMI had a relatively good prognosis compared with gross ETE to the recurrent laryngeal nerve, trachea or esophagus, which was different from the findings of previous studies (11, 20, 23). It is still controversial whether DTC with only gSMI should be downgraded to a lower tumor stage and recurrent risk category. To the best of our knowledge, this is the first meta-analysis to assess the impact of gSMI on outcomes in DTC patients. Compared with patients with no ETE, patients with gSMI had an increased risk of recurrence and LNM. For mortality, 10 year DSS and DM, there were no significant differences between the gSMI and no ETE groups. In contrast with those with maximal ETE, patients with gSMI had a decreased risk of recurrence, mortality, LNM, and DM.

According to ATA guidelines, tumors with gross ETE are categorized into the high-risk group because of the more than 20% structural recurrence rate (10). In our study, the LRR rate of the gSMI group ranged from 5 to 25.9%. These relatively lower LRR rates were mainly attributed to the exclusion of some high-risk recurrent patients in these studies (11, 19, 20). In the Danilovic and Li studies, which included all kinds of DTC cases, the LRR rates of gSMI were 24.6 and 25.9% (17, 18). These data were consistent with the ATA guidelines. Based on the site of tumor invasion, gross ETE can be further divided into three subgroups: invasion only to perithyroidal soft tissue, invasion only to strap muscle and invasion beyond the strap muscles (recurrent laryngeal nerve, trachea, esophagus, skin, or subcutaneous tissues). Some authors have speculated that patients with anterior gETE (i.e., strap muscle) have relatively favorable prognosis compared to those with posterior gETE (i.e., recurrent nerve, trachea, esophagus) (24). We also found that DTC patients with gETE beyond the strap muscle suffered a much higher LRR than the other two groups. A possible reason is that gSMI can be easily resected with negative margins (13). In the future, it may be reasonable that gETE beyond the strap muscle is categorized into an extremely high-risk group in the new recurrence risk stratification system, although further high-quality evidence is needed.

The eighth edition of the AJCC/TNM cancer staging system for DTC was published in 2016 (6). It made a substantial change with regard to the T3 category definition. Because mETE, which is identified only on histological examination, carried much less prognostic importance, the new AJCC/TNM system removed mETE in determining the T category (25). Moreover, T3b was defined as a tumor of any size with gSMI. The 8th edition made clear distinctions of disease with no ETE (T1, T2, T3a), gETE only to the strap muscle (T3b) and gETE beyond the strap muscle (T4) (26). In our study, we found that there was no significant difference between the no ETE and gSMI groups on 10 year DSS. Besides ETE, age, LNM, and DM also play important roles in AJCC system for survival prediction. These factors may be different between “no ETE” and “gross strap muscle invasion” groups, which may explain the similar 10 year DSS between the no ETE and gSMI groups if these factors are adjusted on a multivariate analysis.

Usually, the T stage of tumors is associated with LNM and DM. The invasiveness of tumors represents its severity and differentiation (27). Patients with aggressive tumors are always accompanied by more LNM and early DM (28). Compared with patients with no ETE, patients with gSMI present with more LNM. Maximal ETE was considered an independent risk factor for LNM and DM in contrast with gSMI. This finding in our study suggests that the degree of ETE carries much more prognostic significance for DTC (7).

High heterogeneity with an *I*^2^ > 50% was found in the analysis of LNM (compared with no ETE) and DM (compared with no ETE). Additionally, after the removal of each study from the analysis, similar results were confirmed, and the heterogeneity was not changed significantly. Furthermore, subgroup analysis was performed to explore the source of heterogeneity. In the therapeutic LND subgroup, gSMI increased LNM in comparison with ETE. This finding suggested that clinical LNM was more frequent in patients with gSMI. In the PTC subgroup, we found that gSMI increased DM significantly compared with no ETE. Histopathological types may be correlated with the high heterogeneity in the analysis of DM.

## Strengths and Weaknesses

By performing a meta-analysis with populations from different studies, this is the first study to assess the impact of gSMI on outcomes in DTC patients in a larger study sample and to adjust the results for the presence of some confounding factors. High heterogeneity was found in the analysis of LNM (compared with no ETE) and DM (compared with no ETE) and was compensated by subgroup analysis. The results of LNM (compared with no ETE) and DM (compared with no ETE) should be interpreted with caution because of the limited number of enrolled articles, and further study is needed to confirm the corresponding results.

This meta-analysis has some potential limitations. First, the treatment strategies for DTC patients were different among the enrolled studies. These treatment disparities, such as thyroidectomy, lymph node dissection, radioiodine ablation, or follow-up, might contribute to different patient outcomes. Second, the limited number of studies hindered the implementation of meta-regression analysis and publication bias assessment. The results of the subgroup analysis should be interpreted with caution because of the small number of studies, although heterogeneity was eliminated by subgroup analysis. Third, the retrospective and non-randomized nature of all studies included in the analysis might be considered a source of bias. This provided associative, not causal, evidence, and mandates caution when interpreting these results. In future studies, randomized controlled trials with a higher methodological quality are needed to improve the quality of evidence.

## Conclusion

Patients with gSMI had a higher risk of recurrence and LNM than those without ETE. However, in contrast with maximal ETE, a much better prognosis was observed in DTC patients with only gSMI.

## Data Availability Statement

All datasets generated for this study are included in the article/supplementary material.

## Author Contributions

All authors listed have made a substantial, direct and intellectual contribution to the work, and approved it for publication.

## Conflict of Interest

The authors declare that the research was conducted in the absence of any commercial or financial relationships that could be construed as a potential conflict of interest.
